# Neuroinflammation and Aβ Accumulation Linked To Systemic Inflammation Are Decreased By Genetic PKR Down-Regulation

**DOI:** 10.1038/srep08489

**Published:** 2015-02-17

**Authors:** Anne-Sophie Carret-Rebillat, Clarisse Pace, Sarah Gourmaud, Laura Ravasi, Samantha Montagne-Stora, Sophie Longueville, Marion Tible, Erika Sudol, Raymond Chuen-Chung Chang, Claire Paquet, François Mouton-Liger, Jacques Hugon

**Affiliations:** 1 Inserm UMR-S942, Lariboisiere Hospital, Paris, France; 2 In vivo preclinical imaging facility, IFR 114 - University of Lille, Nord of France, CHU Lille, France; 3 Inserm UMR-S839 Institut du Fer a Moulin and Inserm UMR932, Paris, France; 4 Laboratory of Neurodegenerative Diseases, Department of Anatomy, LKS Faculty of Medicine, The University of Hong Kong, Pokfulam, Hong Kong SAR; 5 Memory Center, and Department of Histology, Lariboisiere Hospital, University of Paris Diderot, AP-HP, Paris, France

## Abstract

Alzheimer's disease (AD) is a neurodegenerative disorder, marked by senile plaques composed of amyloid-β (Aβ) peptide, neurofibrillary tangles, neuronal loss and neuroinflammation. Previous works have suggested that systemic inflammation could contribute to neuroinflammation and enhanced Aβ cerebral concentrations. The molecular pathways leading to these events are not fully understood. PKR is a pro-apoptotic kinase that can trigger inflammation and accumulates in the brain and cerebrospinal fluid of AD patients. The goal of the present study was to assess if LPS-induced neuroinflammation and Aβ production could be altered by genetic PKR down regulation. The results show that, in the hippocampus of LPS-injected wild type mice, neuroinflammation, cytokine release and Aβ production are significantly increased and not in LPS-treated PKR knock-out mice. In addition BACE1 and activated STAT3 levels, a putative transcriptional regulator of BACE1, were not found increased in the brain of PKR knock-out mice as observed in wild type mice. Using PET imaging, the decrease of hippocampal metabolism induced by systemic LPS was not observed in LPS-treated PKR knock-out mice. Altogether, these findings demonstrate that PKR plays a major role in brain changes induced by LPS and could be a valid target to modulate neuroinflammation and Aβ production.

Alzheimer's disease (AD) is a neurodegenerative disorder marked by memory disturbances progressively associated with aphasia, apraxia, agnosia and behavioral symptoms. Currently, there is no cure for the disease and symptomatic treatment includes choline esterase inhibitors and glutamate antagonists. AD is neuropathologically characterized by senile plaques made of the accumulation of Aβ-peptides, neurofibrillary tangles formed by hyperphosphorylated tau protein, synaptic and neuronal losses and neuroinflammation including the presence of activated microglia^1^. The cause of the disease is not known but according to the amyloid cascade hypothesis, the toxicity of Aβ or Aβ oligomers could lead to detrimental consequences for neurons and to neuroinflammation^2^.

Brain inflammation is a key component of the pathological lesions detected in the brain of patients suffering from AD^3^,^4^. Astrocytic and microglial cell reactions are often detected surrounding senile plaques. It has been postulated that neuroinflammation could exacerbate brain lesions leading to synaptic dysfunctions and neuronal degeneration. The Aβ-peptide can trigger microglial cell activation inducing the release of the pro-inflammatory cytokines such as TNFα or IL1-β^5^.

Recent works have suggested that systemic inflammation could exacerbate or even drive neuronal dysfunction associated with dementia and that the kinases PKR and JNK could play a role in these molecular events^6^. Common molecular pathways linking Diabetes Mellitus and AD were recently proposed: mild systemic inflammation could trigger abnormal consequences in the brain including impaired neuronal insulin signalling, synapse degradation and memory disturbances associated with the release of TNFα and IL1-β. Systemic inflammation is also known to modify microglial phenotypes and systemic manipulations of inflammation can improve the disease status by negatively alter this progression^7^. This hypothesis stimulated our interest in determining if pro-inflammatory factors such as the ubiquitous kinase PKR, acting peripherally and in the brain, could contribute to abnormal molecular signals leading to increased neuroinflammation and AD brain lesions during systemic inflammation.

PKR is a pro-apoptotic kinase that controls the initial step of protein translation through the phosphorylation of the eukaryotic initiation factor 2 alpha (eIF2α)^8^. PKR is involved in several cellular pathways including innate immunity and defence against viruses. PKR can sequentially induced cell survival and death pathways^9^. PKR also modulates the synthesis of pro-inflammatory factors via the activation of the NF-κB (nuclear factor κ light-chain-enhancer of activated B cells) pathway after direct interaction with IKKβ (inhibitor of nuclear factor kappa-B kinase subunit beta)^10^. The PKR inhibitor C16 can prevent IL-1β and neuronal apoptosis induced by quinolinic acid administration^11^. PKR is also involved in the control of the inflammasome and HMGB1 (high-mobility group protein B1) release^12^. This kinase is highly expressed in degenerative neurons in AD brains and can be activated in primary neuronal cultures by Aβ^13^,^14^. In addition the levels of phosphorylated PKR are highly increased in the cerebrospinal fluid (CSF) of patients suffering from AD or Mild Cognitive Impairment^15^ and can correlate with the cognitive decline in AD patients^16^.

Since PKR is elevated in AD CSF and brains and can modulate neuroinflammatory signals, we sought to determine if PKR could control brain inflammation and Aβ accumulation after systemic LPS administration in wild-type (WT) mice and PKR^-/-^ mice. Previous studies reported neuroinflammation induction and Aβ-increase after systemic LPS administration^17^. By use of this experimental model, we further investigated whether PKR played a role in triggering the molecular pathways leading to these pathological processes. The results show that PKR genetic down-regulation reduces neuroinflammation and Aβ accumulation.

## Results

### Systemic injection of LPS induces brain PKR phosphorylation

**Figure 1:**
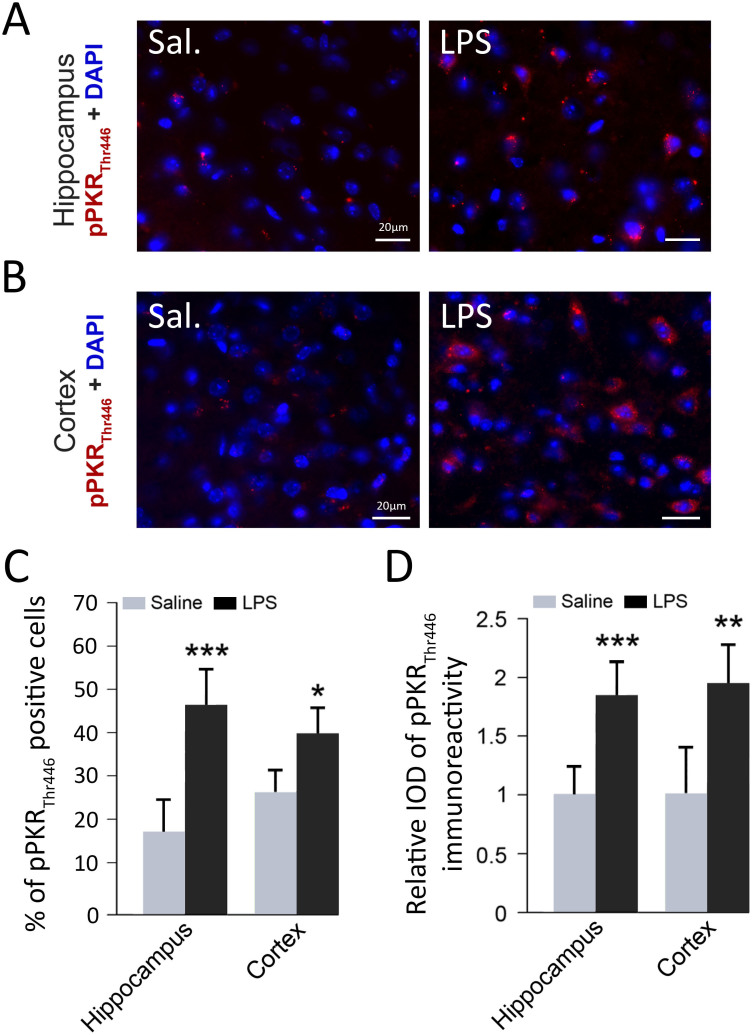
Activation of PKR in WT mice cortex and hippocampus after LPS systemic challenge. Double-labeling in immunofluorescence of DAPI (blue) and pPKR_Thr446_ (red) in hippocampus (A) and frontal cortex (B) sagittal sections of WT mice treated with saline (sal) or LPS. Percentage of pPKR_Thr446_ positive cells (C) and relative IOD (integrated optical density) of pPKR_Thr446_ immunoreactivity (D) are increased in LPS-treated mice. WT [n = 4], *p < 0.05, **p < 0.01, ***p < 0.001.

To assess the consequences of repeated LPS injections on PKR activation, we first performed immunofluorescence analyses of pPKR staining in sagittal slices of WT mice brain exposed to saline or LPS. In WT mice treated with saline solution, we observed a weak cytoplasmic pPKR_Thr446_ staining in hippocampus and cortex (Figure 1A–B). Repeated injections of LPS lead to a dramatic increase of number of pPKR_Thr446_ positive cells (Figure 1C) in both hippocampus and cortex of WT mice. We found a similar increase in hippocampus by evaluating with imageJ software the intensity of staining (Figure 1D). To evaluate the potential pro-apoptotic effects of PKR in this neuroinflammatory model, we have measured by immunoblotting the cleavage by caspase-3 of the nuclear enzyme poly ADP-ribose polymerase (PARP). LPS-induced PKR activation did not lead to a cleavage of PARP protein (Supplementary Figure 1).

### PKR down-regulation prevents hippocampal LPS-induced microglial activation and cytokines production

**Figure 2:**
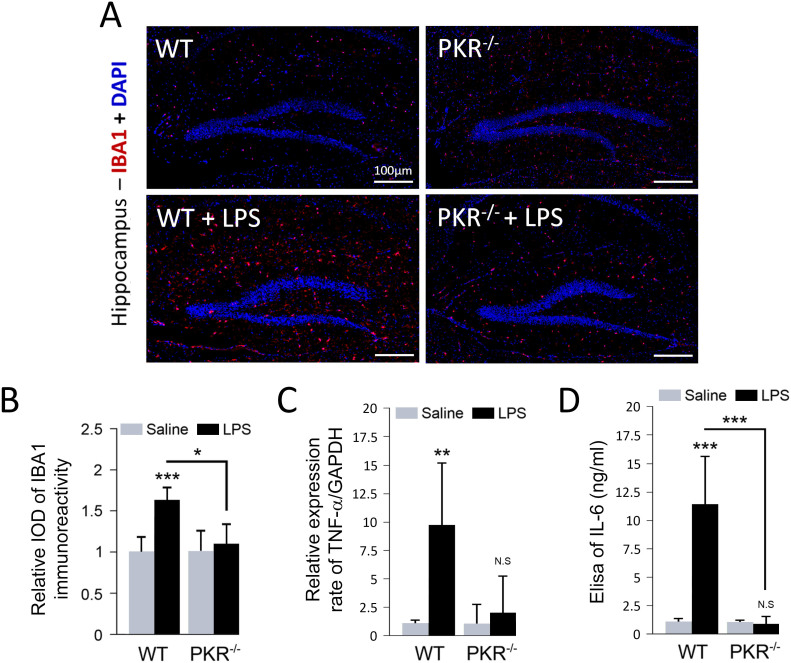
Microglial activation in WT and PKR^-/-^ mice hippocampus. Double-labeling in immunofluorescence of DAPI (blue) and IBA1 (red) in hippocampus sagittal sections of WT and PKR^-/-^ mice treated with saline or LPS (A). Relative IOD (integrated optical density) of IBA1 immunoreactivity in activated microglial cells. LPS induces microglial activation in WT mice. PKR inhibition prevents microglial activation after LPS treatment (B). mRNA level of TNF-α evaluated by qRT-PCR and normalized to GAPDH mRNA levels (C). Brain Il-6 levels in controls or after LPS injection in wild type mice and PKR knock-out mice (D). WT [n = 4], PKR^-/- ^[n = 4], *p < 0.05, **p < 0.01, ***p < 0.001.

Microglia and astrocytes are known to be major sources of neuro-inflammation and are strongly activated by intra-peritoneal LPS injection. Our immunohistofluorescence using a specific marker of microglial cells activation, IBA1 (Ionized calcium-binding adaptor molecule 1) confirmed this finding (Figure 2A). Quantified immunofluorescence analyses of IBA1 positive microglia in the hippocampus, using ImageJ software, revealed a 64% increase of the staining intensity in LPS-treated mice compared to untreated mice (Figure 2B). Next, we sought to understand the role of activated PKR in neuroinflammation by injecting LPS in PKR knock-down mice (PKR^-/-^). PKR^-/-^ mice did not express the functional 69 kDa protein but only at very low level a truncated product of translation (42 kDa) of PKR mRNA devoid of exons 2 and 3 (Supplementary Figure 2).

Analyses of IBA1 staining in hippocampus of LPS-treated PKR^-/-^ mice revealed a significant decrease of microglial activation (−43%) compared to LPS-treated WT mice (Figure 2B). Similar variations were found on astrocytes activation, quantified by enumerating GFAP (glial fibrillary acidic protein) -positive cells in hippocampus (Supplementary Figure 2). Finally, we assessed hippocampal cytokine TNF-α mRNA level by quantitative real time PCR. As expected, the mRNAs level of TNF-α is dramatically increased in LPS-treated mice (+890%). In PKR^-/-^ mice, LPS-induced TNF-α appears less overexpressed (+99%) (Figure 2C). In addition using Elisa, we also found that PKR down regulation abolished the increase of brain IL-6 levels induced by systemic LPS injection (Figure 2D).

### BACE1 upregulation and Aβ production induced by intra-peritoneal LPS injection is reversed in PKR^-/-^ mice

**Figure 3:**
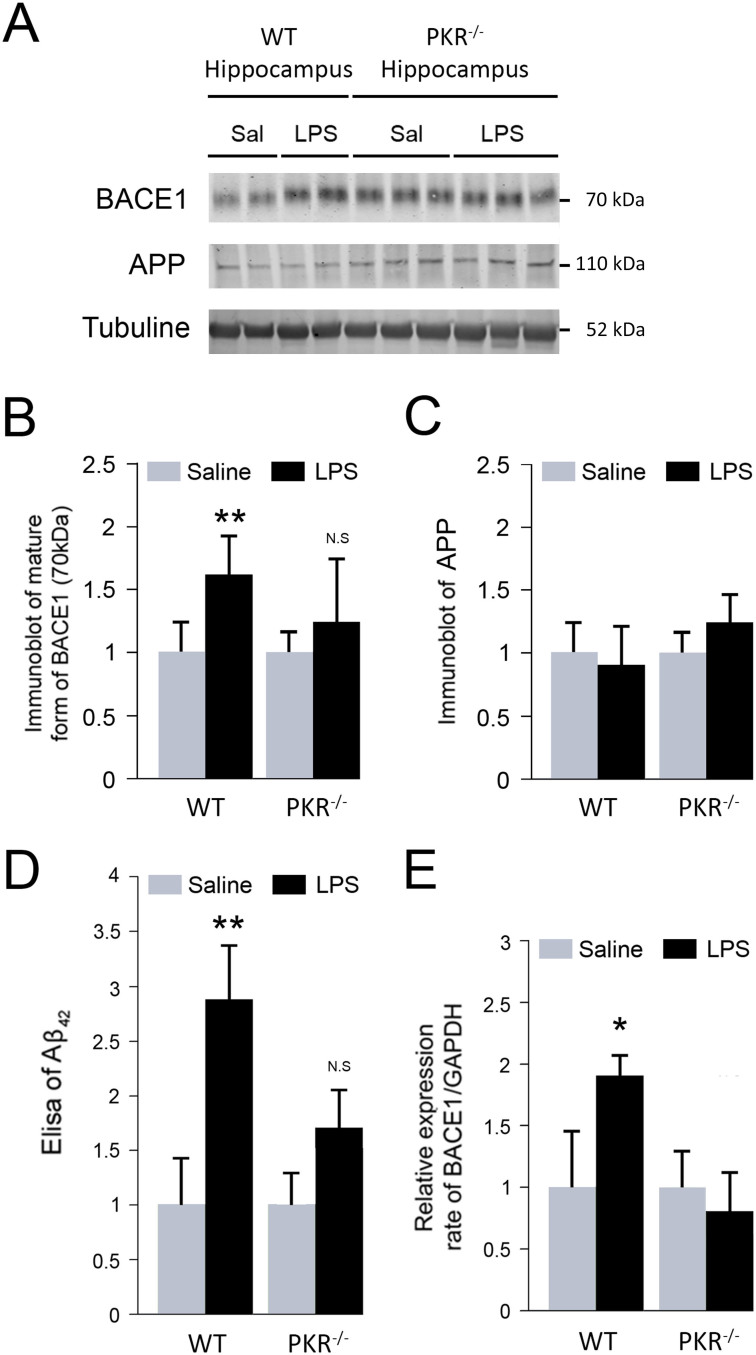
Inhibition of PKR decreases BACE1 levels and Aβ production in mice hippocampus after LPS systemic challenge. Immunoblot analysis (A) and protein levels of mature BACE1 (B) and APP (C) and quantification of Aβ_1–42_ by ELISA (D) in WT and PKR^-/-^ mice treated with saline or LPS. LPS induces BACE1 maturation and Aβ production without altering APP levels in WT mice. PKR inhibition prevents BACE1 maturation and Aβ production after LPS treatment. Transcriptional activity on BACE1 in hippocampus assessed with qRT-PCR and normalized to GAPDH mRNA levels (E). Immunoblots (A) have been cropped in this figure. Full length version of BACE1 and APP blots are available in the supplementary Figure 5. WT [n = 4], PKR^-/-^ [n = 4], **p < 0.01.

Intraperitoneal LPS administration is known to induce a brain Aβ accumulation in WT mice. To confirm this previous observation in our specific model of repeated LPS injection, we first assessed hippocampal APP processing by immunoblotting analyses of mature BACE1 (Figure 3A–B) and APP proteins (Figure 3A and C) and then by evaluating soluble Aβ level with a specific Elisa assay (Figure 3D). Our results are consistent with observations obtained in other LPS models, with a significant increase of mature BACE1 protein level (+58%) and Aβ production (+184%). By contrast, there were no changes in APP protein. To test whether upregulation of BACE1 levels by neuroinflammation occurs only at a translational level, we measured mRNA levels of BACE1 by real-time quantitative PCR. Levels of BACE1 messengers from LPS-treated WT mice were 1.8 fold higher than those of untreated-WT mice (Figure 3E). Interestingly, LPS-treated PKR^-/-^ mice exhibit significantly reduced levels of soluble Aβ and of BACE1 mRNA and protein compared to LPS-treated WT.

As PKR activation could also trigger tau phosphorylation in stress conditions through glycogen synthase kinase -3β (GSK3-β) phosphorylation on tyrosine 216 (pGSK3-β), we performed immunoblot analysis of pGSK3-β, GSK3-β total protein, tau total protein and phosphorylated tau on different sites, such as AT180 (supplementary Figure 4A). Our results reveal a significant increase of pGSK3-β/GSK3-β ratios (+34%) (Supplementary Figure 4B) which is not associated with an increase of tau phosphorylation on AT180 (supplementary Figure 4C), AT8 or AT270 (not shown).

### Increase of activated STAT3 level by systemic LPS administration is PKR dependent

**Figure 4:**
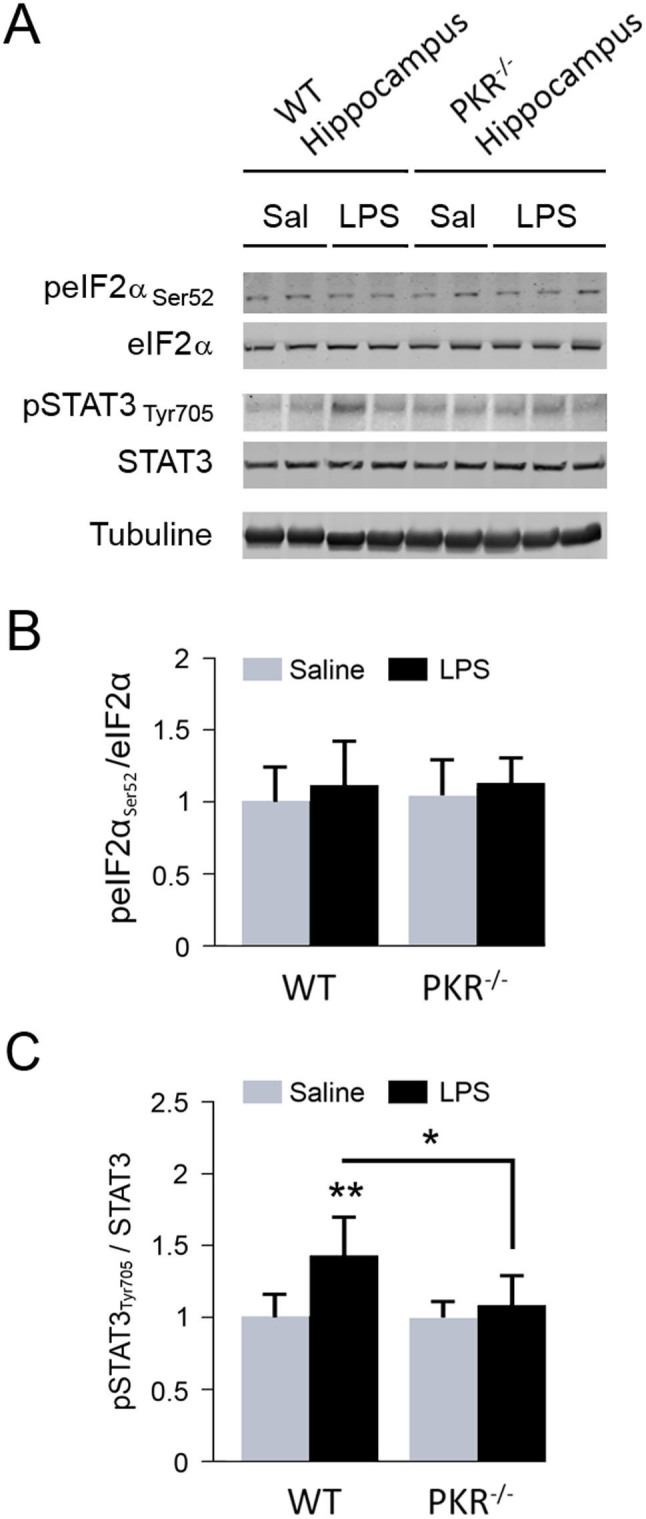
Activation of transcription factor STAT3 after LPS systemic challenge is down-regulated by PKR but not translation initiation factor eIF2α. Immunoblot analysis (A) and corresponding quantification of peIF2α_Ser51_/eIF2α (B) and pSTAT3_Tyr705_/STAT3 (C) ratios in hippocampus of WT and PKR^-/-^ mice treated with saline or LPS. LPS induces STAT3 but not eIF2α activation in WT mice. PKR inhibition prevents STAT3 activation. Cropped immunoblots are presented in this figure. Full length immunoblots of eIF2α (phosphorylated and total forms) and STAT3 (phosphorylated and total forms) are available in the supplementary Figure 6. WT [n = 4], *p < 0.05, **p < 0.01.

Previous *in vitro* researches have shown that stress-induced eIF2α phosphorylation at serine 52 by PKR could increase BACE1 translation and lead to Aβ over-production. Immunoblotting for phosphorylated eIF2α (peIF2α) and eIF2α total protein (Figure 4A) enabled the assessment of the role of the eIF2α activation in LPS-induced amyloidogenic signaling. It showed that peIF2α_Ser52_/eIF2α ratio is not affected by systemic LPS administration (Figure 4B), suggesting an alternative PKR-dependent pathway for BACE1 upregulation. Activation of the transcription factor STAT3 by phosphorylation on tyrosine 705 is able to regulate BACE1 expression in neurons. Immunoblot analysis of pSTAT3_Tyr705_/STAT3 ratio revealed a statistically significant increase (+43%) in LPS-treated WT mice versus saline-injected mice (Figure 4C). It is worth noting that such increase was no longer observed after PKR down regulation.

### PKR inhibition prevents hippocampal hypometabolism after LPS systemic challenge

**Figure 5:**
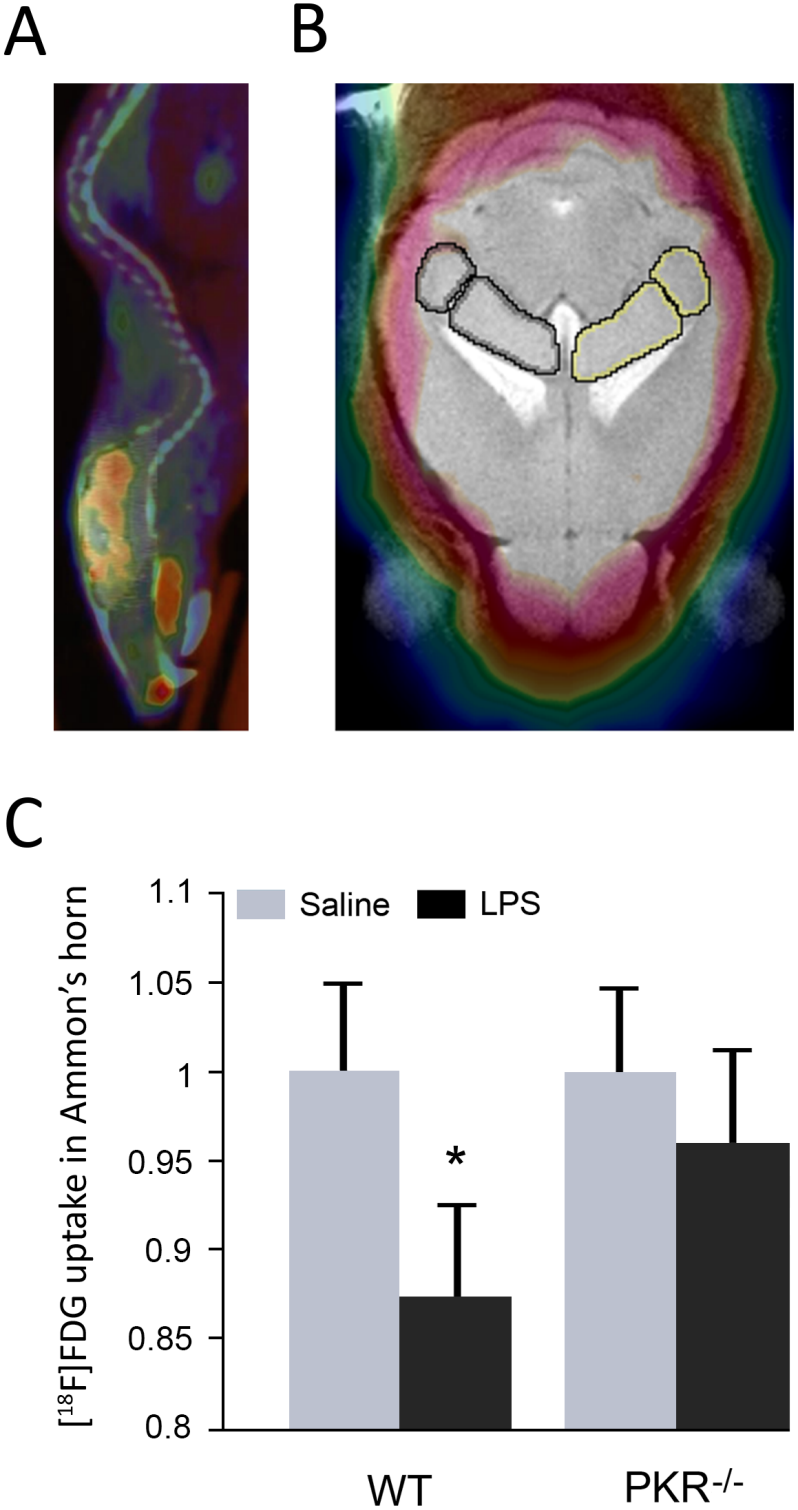
(A) Sagittal view of a co-registered PET-CT whole-body mouse. (B) Coronal view of a co-registered murine cerebral MRI-PET. Regions of interest have been drawn on the hippocampus, bilaterally. (C) Hippocampal, Ammon's horn, in vivo metabolism through [^18^F]FDG-PET.

Motion matrix from the CT to the MR was applied to the PET scan (automatically coregistered to the CT scan) (Figure 5A). Volumes of interest were retreived on the PET scan (Figure 5B) and semi-quantification of regional radiotracer uptake, expressed as a percentage of Injected Dose/g, was normalised to the cerebellar uptake. An average was calculated and the region was then considered as striata. The regional tracer uptake normalized the cerebellar one, was averaged among the mice within each group. We reported in Figure 5 the impairment of metabolism in a subregion of the hippocampus, the Ammon's horn.

In this specific region, the LPS-WT animals had a statistically significant metabolic drop when compared to the controls. However, in the PKR^-/-^ mice, the metabolism of the Ammon's horn did not differ whether the mice were under LPS treatment or not (Figure 5C).

## Discussion

Our results show that systemic inflammation produced by LPS administration can induce neuroinflammation and increased brain Aβ production in wild-type mice. In addition, the genetic invalidation of the kinase PKR in PKR^-/-^ mice leads to the reduction of neuroinflammation and Aβ accumulation in these mice treated with systemic LPS. Although in this model phosphorylated tau was not modified in LPS treated mice as compared to non-treated mice, activated GSK3β, and activated STAT3 were increased in LPS treated mice. PKR down regulation has prevented the increased brain levels of these enzymes in LPS injected mice. Several questions are to be addressed.

Firstly, is there a modification of peripheral inflammation in PKR^-/-^ mice underpinning the reduction of neuroinflammation detected in PKR^-/-^ mice treated with repeated LPS administration? An answer may be found by further assessing mRNA levels of TNF-α or other cytokines (IL-1β, IL-6…) in the spleen or liver of wild-type and PKR^-/-^ injected animals.

A second question is how can PKR control brain inflammation? Previous studies have shown that LPS injection induces the activation of the innate immune system with production of blood cytokines such as TNF-α and IL-1β that are able to enter the brain in regions with weak blood brain barrier or by active transport through the endothelium among several mechanisms^18^. Consequently, cytokines can activate microglial cells that can also release local cytokines and can induce a spreading of neuroinflammation. PKR has been shown to participate in the production of inflammatory signals either by directly activating IKKβ and the NF-κB signaling^19^ or by partially controlling the inflammasome^20^. In our study, TNFα is decreased in injected PKR^-/-^ brain as compared to WT mice suggesting that the invalidation of PKR has reduced the ability of microglial cells to synthesize inflammatory cytokines and transfer neuro-inflammatory signals. This effect is also revealed in the hippocampus by the reduction of activated microglial cells in LPS-injected PKR^-/-^ mice as compared to LPS-injected WT mice. The result showing that brain PKR is activated in LPS-injected WT mice as compared to control mice validate this model of neuroinflammation and strengthen the putative role of PKR signaling in the control of cytokines release and microglial activation.

An additional question is how PKR can control Aβ production in this model? Previous reports have revealed that systemic LPS administration can increase Aβ levels in the brain of injected mice^21^,^22^. Our results have confirmed these results in wild-type mice but have also found that PKR genetic invalidation partially prevented brain Aβ accumulation in PKR^-/-^mice. It is not known if this event is linked to increased Aβ production or reduced Aβ degradation but Aβ accumulation is associated with a clear modification of BACE1 protein levels that could lead to increased Aβ production. There are at least two mechanisms that could link BACE1 and PKR activation. The first one is associated with the peculiar upstream open reading frame of BACE1, which leads to an increased mRNA expression under the control of eIF2α^23^,^24^. Surprisingly, 24 hours after the last LPS administration, eIF2α phosphorylation was not found augmented in WT mice whereas phosphorylated PKR was increased. This could be due to the specific activation of eIF2α phosphatases one day after the last LPS injection. As opposed to what previous authors have observed, a recent paper has shown that inhibition of eIF2α phosphorylation did not alter BACE1 levels and Aβ production in neuronal cultures or in transgenic mice^25^. This finding argues in favor of a PKR-dependent and eIF2α-independent mechanism controlling BACE1 levels in neurons. The second mechanism is associated with the control of BACE1 mRNA synthesis by STAT3^26^. Our results have revealed that brain activated STAT3 levels are increased in LPS-injected wild-type mice and not in LPS-injected PKR^-/-^ mice. A previous data has shown that PKR can control the activation of STAT3 and the triggering of the PKR/STAT3/BACE1 pathway could also explain the features of Aβ accumulation in LPS-treated wild-type and PKR^-/-^ mice. Further studies will have to analyze the process of Aβ degradation in this model as well as the putative involvement of γ-secretase in Aβ accumulation.

A last question is why phosphorylated tau is not modified in the brain of LPS-injected mice. A previous study has shown that LPS administration in 3xTg AD mice exacerbates tau phosphorylation at the AT8 antibody site^27^. In our study although we observed a pGSK3-β increase in LPS-treated mice which was not associated with increased tau phosphorylation at the AT8 site. As already described for eIF2α phosphorylation, one can suggest that specific phosphatases are activated 24 h after the LPS administration and further studies will be needed to determine the time course of brain tau phosphorylation and dephosphorylation in this model. The process of tau phosphorylation could also differ in the brains of wild-type mice and transgenic mice after LPS injection.

Our [^18^F]FDG-microPET study revealed that LPS injection in wild-type mice decreases metabolism in the hippocampus (Amon's Horn) but this effect was not seen in LPS-injected PKR^-/-^ mice. It has been shown previously that sepsis can induce neuroinflammation and a reduction of cerebral metabolism in experimental animals^28^. The authors have demonstrated that in LPS-treated rats, cerebral glucose uptake was reduced in cortical areas and not in the hippocampus but animals received a single LPS injection. The fact that PKR genetic down regulation prevents the modification of neuronal metabolism could be due to the reduced brain neuroinflammation, to the reduction of the observed Aβ accumulation or to the inhibition of pro-apoptotic pathway associated with PKR activation^29^ although cleaved PARP levels, indicative of caspase 3 activation, were not modified in LPS-injected wild-type mice.

There are some limitations in our study. All the assessments were performed only at a single time point, 24 hours after the last LPS injection. Further studies will have to decipher if a recovery phase is observed one or two weeks after the induced systemic inflammation either concerning neuroinflammation or Aβ accumulation. Since LPS induces a sickness syndrome, it is difficult to properly assess behavioral disturbances at the time point of our study but cognitive tests could be performed in the future during the recovery phase after LPS administration. Finally the use of new and specific PKR inhibitors in the future will be needed to determine if these results can be reproduced using a pharmacological approach.

**Figure 6:**
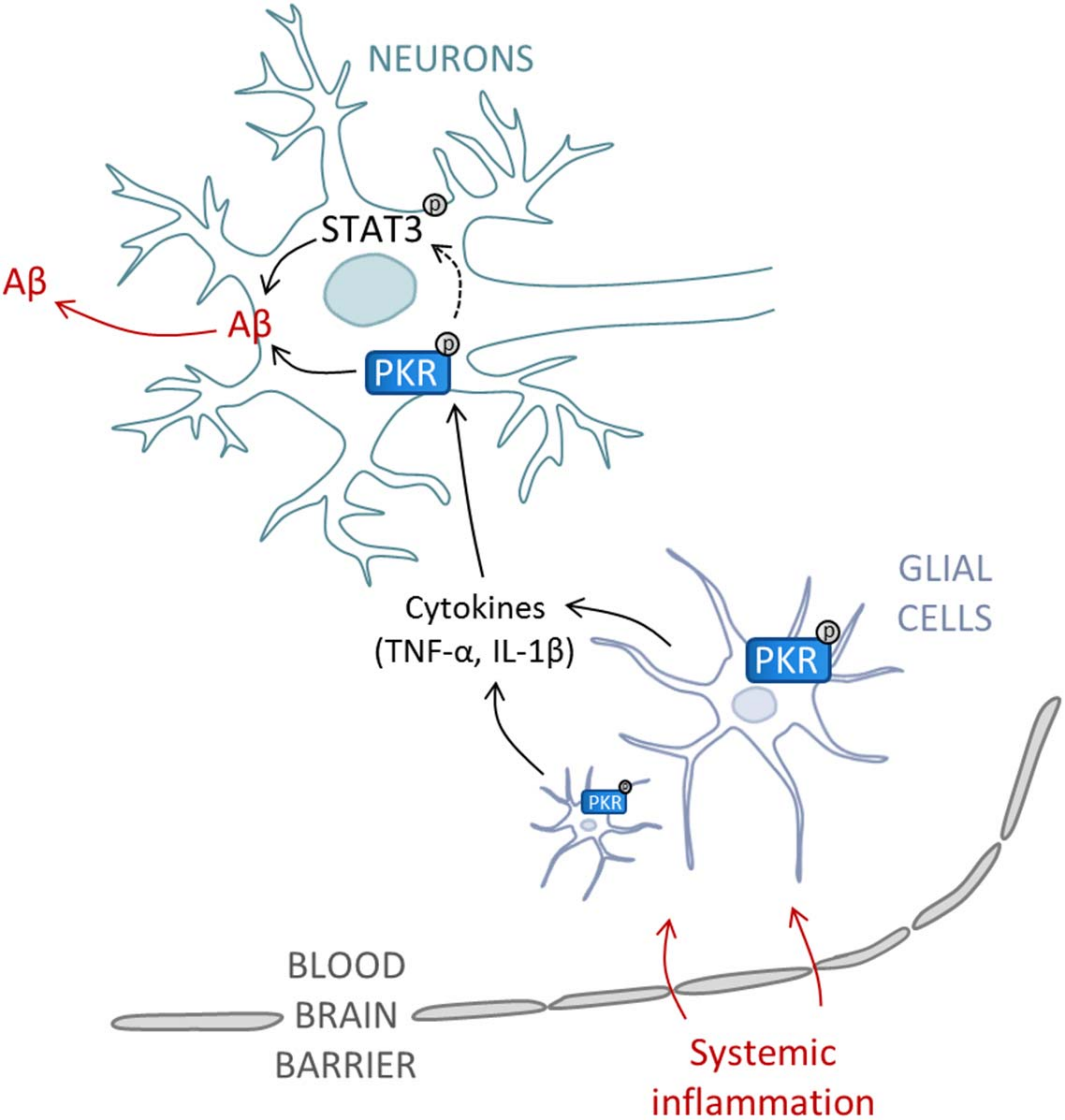
Schematic representation of PKR-dependent Aβ production in brain after systemic inflammation. Peripheral inflammation communicates with the brain through the blood-brain barrier to induce microglial activation and pro-inflammatory cytokines production, involving PKR pathway. Cytokines, including TNFα, activate PKR in neurons leading to Aβ production. Thereof could be controlled by the activation of transcription factor STAT3.

Previous studies have shown that systemic infections can exacerbate the evolution of AD patients^30^,^31^. In this experimental study, we have shown that systemic inflammation can lead to brain PKR activation and Aβ accumulation (Figure 6). In addition, phosphorylated PKR is increased in the brains and CSF of AD or mild cognitive impairment (MCI)-due to AD patients and CSF PKR levels could correlate with cognitive decline. The reason for increased levels of PKR in AD brain is not known but could be associated with cytokine release or Aβ toxicity and also could be exacerbated by systemic infections or inflammation. An earlier data has revealed that PKR activation could negatively control memory formation in experimental animals^32^.

All these findings argue for an early neuroinflammatory process in AD which could be secondarily driven by systemic inflammation and underline the fact that PKR could be a valid new therapeutic target to reduce neuroinflammation and AD brain lesions, to afford neuroprotection and improve memory in affected individuals^33^.

## Methods

### Animals

All experimental protocols used in this study were approved by the Animal Experiments Committee of the INSERM Institut du Fer à Moulin (UMR-S839) and were performed in accordance with the guidelines of the French Agriculture and Forestry Ministry for handling animals (decree 87849, license A75-05-22). C57BL/6J wild type (WT) and PKR knockout (PKR^-/-^) mice (male, 10 weeks old) were used for all experiments. WT mice were purchased from Janvier (Le Genest St Isle, France).

PKR^-/-^ mice, generated by disruption of exons 2 and 3 of the PKR gene^34^ were provided by Dr RCC Chang, The University of Hong Kong, Li Ka Shing Faculty of Medicine, Department of Anatomy, Laboratory of Neurodegenerative Diseases.

All mice were held in a temperature-controlled room under a 12 hours light/dark cycle and had access to food and water *ad libitum*. Animal experiments were performed in accordance with the guidelines of the French Agriculture, Food and Forestry Ministry for handling animals (decree 87849, license A75-05-22).

Mice received daily intraperitoneal (i.p) injections of either saline or LPS from *Escherichia coli* 0111:B4 (Millipore, Molsheim, France), 1 mg/kg for 3 days. Twenty-four hours after the last injection, 20 mice were taken to the *in vivo* imaging facility whereas all others were deeply anesthetized with a lethal dose of pentobarbital and intracardially perfused with cold PBS. Brains were then collected in ice, dissected and fixed in 4% paraformaldehyde for immunohistochemistry or immediately frozen in liquid nitrogen for immunoblotting, ELISA or quantitative RT-PCR.

### Immunoblotting and ELISA

Cortex and hippocampus were homogenized and sonicated in a radioimmunoprecipitation assay buffer (RIPA buffer) containing 10 mM NaPi pH 7.8, 59 mM NaCl, 1% Triton, 0.5% DOC, 0.1% SDS, 10% glycerol and extemporaneous addition of protease inhibitor cocktail (Roche, Penzberg, Germany), 1 mM Na3VO4 and 0.1 μM calyculin A (Sigma, St Louis, MO) as phosphatase inhibitors. Tissues were then centrifuged at 15 000 g for 10 minutes and protein concentration is determined with Micro BCA Protein Assay Reagent Kit (Thermo scientific, Cergy-Pontoise, France) using manufacturer's protocol.

For immunoblotting, after denaturation (96°C, 5 minutes, in β-mercaptoethanol with 150 g/L SDS, 0.3 M Tris-HCl pH 6.8, 25% glycerol and bromophenol blue), protein samples (25 to 50 μg) were separated on Tris-glycine polyacrylamide gels (Bio-Rad, Nanterre, France) and transferred onto nitrocellulose membranes (GE Healthcare, Chalfont St. Giles, UK) in 25 mM Tris pH 8.3, 200 mM glycine and 20% ethanol. Membranes were incubated 30 minutes in blocking buffer (1 X TBS with 5% milk), and then overnight at 4°C with primary antibodies. Rabbit anti-BACE1 (Santa Cruz, Danvers, MA, USA), rabbit anti-APP (Cell Signaling, Beverly, MA, USA), mouse anti-phosphorylated GSK3β_Tyr216_ (pGSK3β_Tyr216_) (BD Transduction Laboratories, San Jose, CA, USA), mouse anti-GSK3β (Cell Signaling), mouse AT180 (Thr231/Ser235) (Thermo Scientific), mouse anti-Tau (Thermo Scientific), rabbit anti-pSTAT3_Tyr705_ (Cell Signaling), rabbit anti-STAT3 (Cell Signaling), rabbit anti-actin (Sigma, St. Louis, MO) and mouse anti-tubulin (Santa Cruz) were used as primary antibodies. IR Dye 700DX conjugated anti-mouse IgG and IR Dye 800 CW conjugated anti-rabbit IgG (Rockland Immunochemical Inc., Gilbertsville, PA, USA) were used as secondary antibodies. Protein bands were revealed by use of the Odyssey imaging system (Li-Cor Biosciences, Lincoln, NE, USA) and quantified with the Multigauge software (Fuji-film, Tokyo, Japan).

Aβ_1–42_ levels were quantified using a mouse colorimetric Aβ_1–42_ ELISA kit (Covance Inc., Princeton, NJ, USA) following the manufacturer's instructions. Optical absorbance was detected using a 96 well plate reader at 450 nm. Aβ levels were calculated from a standard curve.

IL-6 levels were quantified using a mouse colorimetric IL-6 ELISA kit (Signosis Inc.,Santa Clara, CA, USA) following the manufacturer's instructions. Optical absorbance was detected using a 96 well plate reader at 450 nm. IL-6 levels were calculated from a third-order polynomial regression curve.

### Immunohistofluorescence

After fixation in 4% paraformaldehyde, brains were incubated in 30% sucrose, frozen in Jung tissue medium (Leica, Nanterre, France) and sectioned using a cryostat. Sagittal sections (10 μm) were washed in PBS with 0.25% gelatin and 25% Triton and incubated at 4°C for 24 h with rabbit anti-ionized binding molecule adaptor 1 (Iba1) (Wako, Osaka, Japan) and rabbit anti-pPKR_Thr446_ (Abcam, Cambridge, UK) and at room temperature for 2 h with secondary antibodies donkey anti-rabbit Cy3 (Jackson Laboratory, Bar Harbor, Maine, USA). Standard epifluorescence images were acquired on a Leica DMRD microscope using a high resolution camera (Coolsnap HQ). The Metamorph software (Roper Scientific, Sarasota, FL, USA) was used for image acquisition. All quantitative image analyses were performed by using NIH ImageJ software, as previously described^35^. Quantification was limited to the areas corresponding to the cortex and hippocampus. DAPI and the cyanine 3 pictures were both background corrected using the rolling ball method. A threshold was then chosen using the ‘Auto threshold’ function of ImageJ. Cells were subsequently evaluated by defining a region of interest and by running the ‘Analyze particles’ imageJ function. Afterward, quantification of positive cells was performed using the Colocalization plug-in.

### Quantitative RT-PCR

Total RNA was isolated using TRIzol reagent (Invitrogen, Carlsbad, CA, USA) from cortex and hippocampus. Transcriptor First Strand cDNA Synthesis kit (Roche) with a combination of random hexamers and oligo(dT) priming to avoid 3′-bias in the cDNAs was used to synthesize the first strand cDNA from samples with an equal amount of total RNA (1 μg), according to the manufacturer's instructions. cDNA samples were forwarded to amplification with Real Time ready Assays (Roche) using LightCycler®96 Instrument (Roche). Each assay included gene specific primers for TNFα (tumor necrosis factor, alpha), BACE-1 (beta-site APP cleaving enzyme 1) and GAPDH (glyceraldehydes-3-phosphate dehydrogenase) and a Universal ProbeLibrary (UPL) Probe, which was a short FAM-labeled (6-carboxyfluorescein) hydrolysis probe containing locked nucleic acid (LNA) (Roche). Mus Musculus BACE1 primers [forward 5′-AAGCTGCCGTCAAGTCCAT-3′ and reverse 5′- CTGCTCCCCTAGCCAAAAG-3′], TNFα primers [forward 5′-TCTTCTCATTCCTGCTTGTGG-3′ and reverse 5′-GGTCTGGGCCATAGAACTGA-3′] and GAPDH primers [forward 5′-AGCTTGTCATCAACGGGAAG-3′ and reverse 5′-TTTGATGTTAGTGGGGTCTCG-3′] were used. cDNA amplification was carried out as follows: denaturation at 95°C for 10 seconds, followed by 45 cycles of denaturation at 95°C for 10 seconds and primer annealing-extension step at 60°C for 30 seconds, ending with a cooling step at 37°C for 30 seconds. All assays were performed in triplicates. Relative levels and gene copy numbers were calculated (normalized to GAPDH) using the previously described deltaCp method^36^.

### [^18^F]FDG *in vivo* imaging

Eleven C57BL/6J wild type (WT) and nine PKR knockout (PKR^-/-^) mice were taken to imaging facility the night before imaging and kept at room temperature with free access to food and water up to 6 hours prior to radiotracer injection when food was no longer accessible. Positron emission tomography scans were performed in a microPET (Inveon, Siemens). A bolus injection of [^18^F]FDG (13+/-1.5 MBq; 150 μl in volume) was administered intraperitoneally while the animal was conscious. After injection, the mouse was returned to its cage to allow for biodistribution for approximately 45 min. Then, the mouse was anesthetized with isoflurane (5% for induction and 1,5% for maintenance in 100% O_2_ at a flow rate of 1 L/min) using a nose cone and placed in a prone position on the platform of the scanner. With the help of a laser alignment device attached to the scanner, the mouse was positioned so that the center of the field corresponded to the brain. A CT scan (80 KV and 500 mA) was run right before the mouse moved into the PET field of view. PET scanning was initiated at 60 min after radiotracer injection. Total scanning duration was 15 min. For co-registration purposes, a 7 T T2-weighted magnetic resonance imaging C57BL/6J brain scan was used to improve cerebral regional delineation on brain PET imaging. Data from the scanner were formatted into 3 frames, 2D-OSEM reconstructed and corrected for scatter and attenuation. Counts detected by the scanner were converted into MBq/mL by use of Inveon Research Workflow (IRW version 3.0, Siemens). This software enables multimodality imaging coregistration and allows manual drawing of volumes of interest (VoIs). MR spatial resolution allows a rather precise identification of several cerebral regions so we drew on the hippocampus on both hemispheres, as shown on the figure 5b. A background VoI was also drawn outside the mouse body. MR whole brain scan was manually coregistered to the CT-scan.

### Statistical Analysis

All experiments were performed independently at least three times. All data were normalized and analyzed using the StatView software (Fuji-film). The paired sign test or Student's *t*-test was used to compare the experimental and control groups. Results were considered significant for a value of p < 0.05 using the student test.

## Supplementary Material

Supplementary Information Supplementary Information

## References

[b1] Duyckaerts C., Delatour B. & Potier M. C. Classification and basic pathology of Alzheimer disease. Acta Neuropathol 118, 5–36 (2009). 19381658 10.1007/s00401-009-0532-1

[b2] Hardy J. & Selkoe D. J. The amyloid hypothesis of Alzheimer's disease: progress and problems on the road to therapeutics. Science 297, 353–356 (2002). 12130773 10.1126/science.1072994

[b3] McGeer E. G. & McGeer P. L. Neuroinflammation in Alzheimer's disease and mild cognitive impairment: a field in its infancy. J Alzheimers Dis 19, 355–361 (2010). 20061650 10.3233/JAD-2010-1219

[b4] Zotova E., Nicoll J. A., Kalaria, R., Holmes, C. & Boche, D. Inflammation in Alzheimer's disease: relevance to pathogenesis and therapy. Alzheimers Res Ther 2, 1 (2010). 20122289 10.1186/alzrt24 PMC2874260

[b5] Khandelwal P. J., Herman A. M. & Moussa C. E. Inflammation in the early stages of neurodegenerative pathology. J Neuroimmunol 238, 1–11 (2011). 21820744 10.1016/j.jneuroim.2011.07.002 PMC3176984

[b6] De Felice F. G. & Ferreira S. T. Inflammation, defective insulin signaling, and mitochondrial dysfunction as common molecular denominators connecting type 2 diabetes to Alzheimer disease. Diabetes 63, 2262–2272 (2014). 24931033 10.2337/db13-1954

[b7] Perry V. H., Nicoll J. A. & Holmes C. Microglia in neurodegenerative disease. Nat Rev Neurol 6, 193–201 (2010). 20234358 10.1038/nrneurol.2010.17

[b8] Marchal J. A. *et al.* The impact of PKR activation: from neurodegeneration to cancer. FASEB J 28, 1965–1974 (2014). 24522206 10.1096/fj.13-248294

[b9] Donze O., Deng J., Curran J., Sladek R., Picard D. & Sonenberg N. The protein kinase PKR: a molecular clock that sequentially activates survival and death programs. EMBO J 23, 564–571 (2004). 14749731 10.1038/sj.emboj.7600078 PMC1271809

[b10] Bonnet M. C., Weil R., Dam E., Hovanessian A. G. & Meurs E. F. PKR stimulates NF-kappaB irrespective of its kinase function by interacting with the IkappaB kinase complex. Mol Cell Biol 20, 4532–4542 (2000). 10848580 10.1128/mcb.20.13.4532-4542.2000 PMC85837

[b11] Tronel C., Page G., Bodard S., Chalon S. & Antier D. The specific PKR inhibitor C16 prevents apoptosis and IL-1beta production in an acute excitotoxic rat model with a neuroinflammatory component. Neurochem Int 64, 73–83 (2014). 24211709 10.1016/j.neuint.2013.10.012

[b12] Lu B. *et al.* Novel role of PKR in inflammasome activation and HMGB1 release. Nature 488, 670–674 (2012). 22801494 10.1038/nature11290 PMC4163918

[b13] Chang R. C., Suen K. C., Ma C. H., Elyaman W., Ng H. K. & Hugon J. Involvement of double-stranded RNA-dependent protein kinase and phosphorylation of eukaryotic initiation factor-2alpha in neuronal degeneration. J Neurochem 83, 1215–1225 (2002). 12437593 10.1046/j.1471-4159.2002.01237.x

[b14] Chang R. C., Wong A. K., Ng H. K. & Hugon J. Phosphorylation of eukaryotic initiation factor-2alpha (eIF2alpha) is associated with neuronal degeneration in Alzheimer's disease. Neuroreport 13, 2429–2432 (2002). 12499843 10.1097/00001756-200212200-00011

[b15] Mouton-Liger F. *et al.* Increased cerebrospinal fluid levels of double-stranded RNA-dependant protein kinase in Alzheimer's disease. Biol Psychiatry 71, 829–835 (2012). 22281122 10.1016/j.biopsych.2011.11.031

[b16] Dumurgier J. *et al.* Cerebrospinal fluid PKR level predicts cognitive decline in Alzheimer's disease. PLoS One 8, e53587 (2013). 23320095 10.1371/journal.pone.0053587 PMC3539966

[b17] Sheng J. G., Bora S. H., Xu G., Borchelt D. R., Price D. L. & Koliatsos V. E. Lipopolysaccharide-induced-neuroinflammation increases intracellular accumulation of amyloid precursor protein and amyloid beta peptide in APPswe transgenic mice. Neurobiol Dis 14, 133–145 (2003). 13678674 10.1016/s0969-9961(03)00069-x

[b18] Schedlowski M., Engler H. & Grigoleit J. S. Endotoxin-induced experimental systemic inflammation in humans: a model to disentangle immune-to-brain communication. Brain Behav Immun 35, 1–8 (2014). 24491305 10.1016/j.bbi.2013.09.015

[b19] Bonnet M. C., Daurat C., Ottone C. & Meurs E. F. The N-terminus of PKR is responsible for the activation of the NF-kappaB signaling pathway by interacting with the IKK complex. Cell Signal 18, 1865–1875 (2006). 16600570 10.1016/j.cellsig.2006.02.010

[b20] Yim H. C. & Williams B. R. Protein kinase R and the inflammasome. J Interferon Cytokine Res 34, 447–454 (2014). 24905201 10.1089/jir.2014.0008

[b21] Kahn M. S. *et al.* Prolonged elevation in hippocampal Abeta and cognitive deficits following repeated endotoxin exposure in the mouse. Behav Brain Res 229, 176–184 (2012). 22249135 10.1016/j.bbr.2012.01.010

[b22] Krstic D. *et al.* Systemic immune challenges trigger and drive Alzheimer-like neuropathology in mice. J Neuroinflammation 9, 151 (2012). 22747753 10.1186/1742-2094-9-151 PMC3483167

[b23] Mouton-Liger F. *et al.* Oxidative stress increases BACE1 protein levels through activation of the PKR-eIF2alpha pathway. Biochim Biophys Acta 1822, 885–896 (2012). 22306812 10.1016/j.bbadis.2012.01.009

[b24] O'Connor T. *et al.* Phosphorylation of the translation initiation factor eIF2alpha increases BACE1 levels and promotes amyloidogenesis. Neuron 60, 988–1009 (2008). 19109907 10.1016/j.neuron.2008.10.047 PMC2667382

[b25] Sadleir K. R., Eimer W. A., Kaufman R. J., Osten P. & Vassar R. Genetic Inhibition of Phosphorylation of the Translation Initiation Factor eIF2alpha Does Not Block Abeta-Dependent Elevation of BACE1 and APP Levels or Reduce Amyloid Pathology in a Mouse Model of Alzheimer's Disease. PLoS One 9, e101643 (2014). 24992504 10.1371/journal.pone.0101643 PMC4081565

[b26] Wen Y. *et al.* Transcriptional regulation of beta-secretase by p25/cdk5 leads to enhanced amyloidogenic processing. Neuron 57, 680–690 (2008). 18341989 10.1016/j.neuron.2008.02.024 PMC2329816

[b27] Kitazawa M., Oddo S., Yamasaki T. R., Green K. N. & LaFerla F. M. Lipopolysaccharide-induced inflammation exacerbates tau pathology by a cyclin-dependent kinase 5-mediated pathway in a transgenic model of Alzheimer's disease. J Neurosci 25, 8843–8853 (2005). 16192374 10.1523/JNEUROSCI.2868-05.2005 PMC6725603

[b28] Semmler A. *et al.* Sepsis causes neuroinflammation and concomitant decrease of cerebral metabolism. J Neuroinflammation 5, 38 (2008). 18793399 10.1186/1742-2094-5-38 PMC2553764

[b29] Gil J. & Esteban M. The interferon-induced protein kinase (PKR), triggers apoptosis through FADD-mediated activation of caspase 8 in a manner independent of Fas and TNF-alpha receptors. Oncogene 19, 3665–3674 (2000). 10951573 10.1038/sj.onc.1203710

[b30] Holmes C., Cunningham C., Zotova E., Culliford D. & Perry V. H. Proinflammatory cytokines, sickness behavior, and Alzheimer disease. Neurology 77, 212–218 (2011). 21753171 10.1212/WNL.0b013e318225ae07 PMC3136056

[b31] Holmes C. *et al.* Systemic inflammation and disease progression in Alzheimer disease. Neurology 73, 768–774 (2009). 19738171 10.1212/WNL.0b013e3181b6bb95 PMC2848584

[b32] Zhu P. J. *et al.* Suppression of PKR promotes network excitability and enhanced cognition by interferon-gamma-mediated disinhibition. Cell 147, 1384–1396 (2011). 22153080 10.1016/j.cell.2011.11.029 PMC3569515

[b33] Hugon J., Paquet C. & Chang R. C. Could PKR inhibition modulate human neurodegeneration? Expert Rev Neurother 9, 1455–1457 (2009). 19831834 10.1586/ern.09.92

[b34] Yang Y. L. *et al.* Deficient signaling in mice devoid of double-stranded RNA-dependent protein kinase. EMBO J 14, 6095–6106 (1995). 8557029 10.1002/j.1460-2075.1995.tb00300.x PMC394734

[b35] Schmuck M. *et al.* Automatic counting and positioning of 5-bromo-2-deoxyuridine (BrdU) positive cells in cortical layers of rat brain slices. Neurotoxicology 43, 127–133 (2014). 24572144 10.1016/j.neuro.2014.02.005

[b36] Pfaffl M. W. A new mathematical model for relative quantification in real-time RT-PCR. Nucleic Acids Res 29, e45 (2001). 11328886 10.1093/nar/29.9.e45 PMC55695

