# Supplementary material for: Neuroinflammation and Aβ Accumulation Linked To Systemic Inflammation Are Decreased By Genetic PKR Down-Regulation
Source: Sci Rep. 2015 Feb 17;5:8489. doi: 10.1038/srep08489 (PMC4330547; doi:10.1038/srep08489)
Supplement: Supplementary Information [file srep08489-s1.docx]

Supplementary Information

**NEUROINFLAMMATION AND Aβ ACCUMULATION LINKED TO SYSTEMIC INFLAMMATION ARE DECREASED BY GENETIC PKR DOWN-REGULATION**

Anne-Sophie Carret-Rebillat^1^, Clarisse Pace^1^, Sarah Gourmaud^1^, Laura Ravasi^2^, Samantha Montagne-Stora^1^, Sophie Longueville^3^, Marion Tible^1^, Erika Sudol^1^, Raymond Chuen-Chung Chang^4^, Claire Paquet^1,5^, François Mouton-Liger^1#^, Jacques Hugon^1,5#^.

**
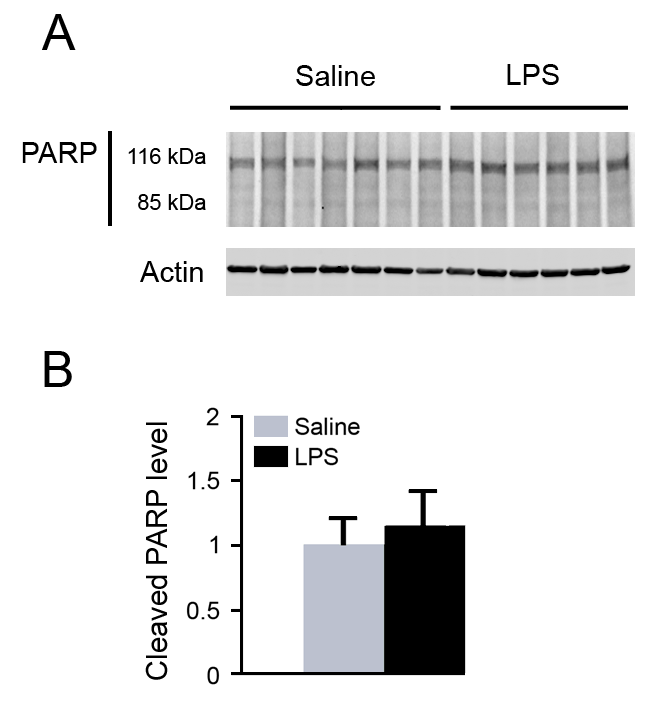
**

**Supplementary Figure 1:** The 85 kDa fragment of cleaved PARP, a marker of apoptosis, is not detected in WT mice cortex after LPS systemic challenge. Immunoblot analysis (A) and protein levels (B) of total (116 kDa) and cleaved (85 kDa) PARP in WT mice treated with saline or LPS [n=6]

**
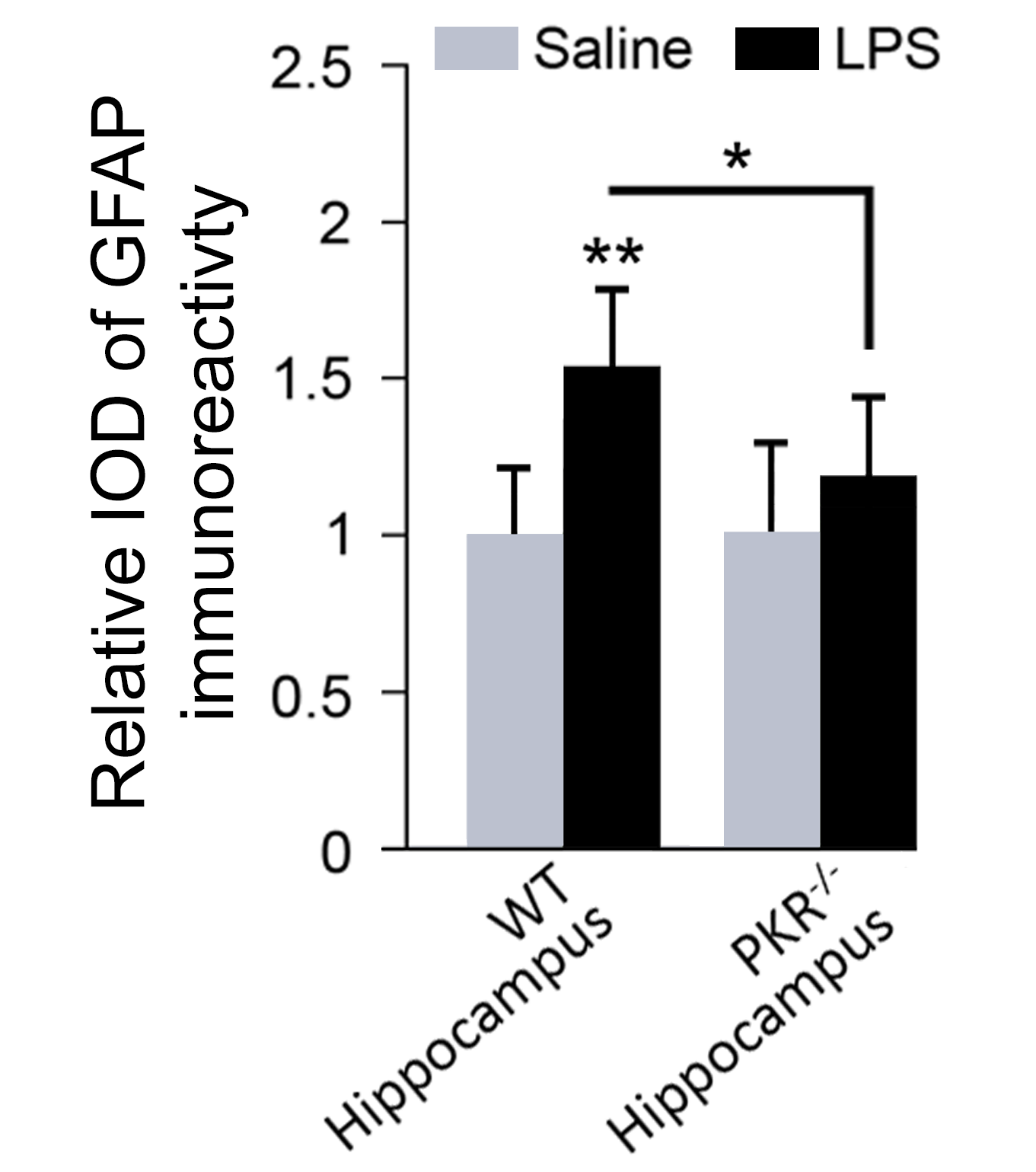
**

**Supplementary Figure 2:** LPS induces astrocyte activation in WT mice but not in PKR^-/-^ mice hippocampus. Astrocyte activation is evaluated by GFAP staining in immunohistofluorescence. WT [n=4], PKR^-/-^ [n=4], *p<0.05, **p<0.01

**
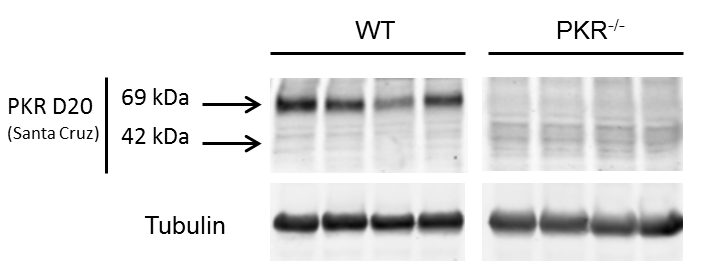
**

**Supplementary Figure 3:** Immunoblot analysis of PKR in WT and PKR^-/-^ mice hippocampus. Expression of total PKR (69 kDa) in WT mice and invalidated truncated form of PKR (42 kDa) in PKR^-/-^ revealed by specific PKR antibody targeting the N terminal domain.


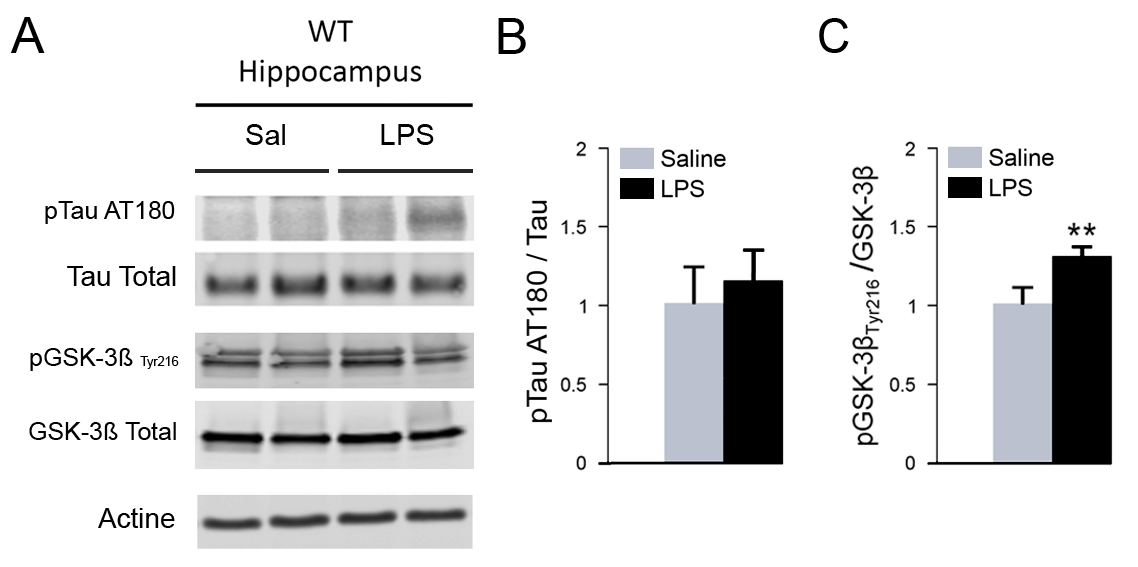


**Supplementary Figure 4:** Immunoblot analysis (A) and corresponding quantification (B) of phosphorylation of GSK3β (pGSK3β_Tyr216_/ GSK3β ratio) and Tau (pTauAT180/Tau) in WT mice hippocampus after LPS systemic challenge or saline injection (sal). WT [n=6], **p<0.01

**Supplementary Figure 5:** Uncropped immunblots from Figure 3A

**Supplementary Figure 6:** Uncropped immunblots from Figure 4A
